# Case Report: Endocrine, immune and disease dynamics in a patient with rheumatoid arthritis during flare and medication change

**DOI:** 10.3389/fimmu.2025.1491475

**Published:** 2025-05-08

**Authors:** Lennart Seizer, Johanna M. Gostner, Christoph Garbers, Melina Licht, Sebastian Sager, Andreas Brandl, Christian Schubert

**Affiliations:** ^1^ Department of Child and Adolescent Psychiatry, Psychosomatics and Psychotherapy, University Hospital of Tübingen, Tübingen, Germany; ^2^ Department of Psychiatry, Psychotherapy, Psychosomatics and Medical Psychology, Medical University of Innsbruck, Innsbruck, Austria; ^3^ Institute of Medical Biochemistry, Biocenter, Medical University of Innsbruck, Innsbruck, Austria; ^4^ Institute of Clinical Biochemistry, Hannover Medical School, Hannover, Germany; ^5^ Faculty of Mathematics, Otto-von-Guericke University Magdeburg, Magdeburg, Germany; ^6^ Conservative and Rehabilitative Orthopedics, Department of Sport and Health Sciences, Technical University of Munich, Munich, Germany

**Keywords:** rheumatoid arthritis, flare, time series, cortisol, neopterin, interleukin-6, orosomucoid-2, integrative single-case study

## Abstract

**Objective:**

Rheumatoid arthritis (RA) is a chronic autoimmune disease of mostly unknown etiology and pathophysiology. In this integrative single-case study on a patient with RA, we had the unique opportunity to closely monitor the individual dynamics of endocrine, immune and disease variables during a naturally occurring flare-up and subsequent medication change.

**Methods:**

The 59-year-old female RA patient collected her entire urine over 30 days in 12-h intervals (60 consecutive measurements in total). Subsequently, cortisol, interleukin-6 (IL-6), orosomucoid-2 (ORM-2), neopterin and creatinine levels were determined in the urine samples. Further, each morning and evening, the patient completed the DIARI, a set of questionnaires on variables such as perceived pain, perceived RA disease activity and emotional states. Once a week, the patient was interviewed online and had an appointment with her rheumatologist, in which several indices of RA disease activity were determined: SDAI, CDAI and DAS28. From these data various time series were constructed for statistical analysis.

**Results:**

RA disease state increased from low to high activity during the first 12 study days. Thereupon, the medication was changed, which proved effective in reducing RA disease activity. However, the levels of urinary neopterin, urinary IL-6 and urinary ORM-2 did not show any response, neither to the increasing disease activity nor the medication change. The patient’s daily reports on pain, RA disease activity, emotional states and body temperature, however, mirrored the course of the rheumatologic indices.

**Conclusion:**

This integrative single-case study clearly demonstrated the importance of process analysis for the evaluation of therapeutic measures in RA. In the studied patient, urinary levels of neopterin, IL-6 and ORM-2 were not found to be appropriate biomarkers of short-term fluctuations in RA disease activity. Instead, the results reported by the patient proved to be a useful tool for ambulatory and longitudinal monitoring of RA.

## Introduction

Rheumatoid arthritis (RA) is a chronic autoimmune disease that includes synovial inflammation and hyperplasia, autoantibody production, cartilage and bone destruction as well as systemic features including cardiovascular, pulmonary, skeletal and psychiatric disorders. The RA disease course is often characterized by fluctuations in disease activity with the sudden re-expression or enhancement of disease pathogenic processes, termed *flare*. For deeper insights into the concept of flares in RA see Bozzalla-Cassione et al. (1). While biological processes during active RA are well known, there is little insight into both the pathodynamics that accompany the transition from remission to flare-up and whether remission in RA refers to the complete restoration of normal body functions (restitutio ad integrum) or to active regulatory mechanisms which keep pathologic processes in check (balanced homeostasis) (1). Further, despite significant progress in the treatment of RA, 20–30% of patients remain refractory to the state-of-the-art therapy (2). Thus, key points in the European Alliance of Associations for Rheumatology’s (EULAR) research agenda contain the monitoring of therapeutic drug use and courses of disease to elucidate molecular pathways in treatment responses and identify associated biomarkers (2).

This paper presents the results of an integrative single-case study designed to advance understanding of the psychoneuroimmunological mechanisms in RA and therapy, conducted under highly ecologically valid conditions (“life as it is lived”) (3). The studied individual collected her entire urine over a period of one month at 12-h intervals, completed questionnaires twice a day, and participated in weekly online in-depth interviews. For the investigation of biopsychosocial dynamics under everyday life conditions, the collection of urine has several advantages: 1) Urine can continuously and cumulatively be collected by the individual under study, 2) the content concentration of urine is only minimally regulated by internal mechanisms, 3) many urinary biomarkers of interest are directly proportional to their serum levels (e.g., cortisol, neopterin, IL-6), 4) the concentration of biomarkers in urine remains stable even with prolonged storage, and 5) urine collection is non-invasive, minimizing psychological stress associated with sample collection (4–9).

In addition to daily urine collection and questionnaires, as well as weekly interviews, the patient had weekly appointments with her rheumatologist for RA activity assessment including blood sampling. Such N-of-1 designs have been recommended in clinical care research since they focus on individual, not average, responses to treatment and adequately handle within-subject variation (4, 10, 11). In the present study, the patient coincidentally experienced an RA flare-up, which offered a unique possibility to investigate the emergence and termination of this flare with high temporal resolution of disease-related parameters under naturalistic conditions.

## Methods

### Study design

This integrative single-case study (3) on a female RA patient is part of a larger trial on personalized therapy in RA [PETRA; (12)]. Shortly before the start of the study, the patient was given a thorough physical examination and psychological assessment. Then, she collected her entire urine for 30 days in 12-h intervals from approx. 8 a.m. to 8 p.m. (day), and approx. 8 p.m. to 8 a.m. (night) using polyethylene urine containers (total of 60 12h-intervals). Upon collection, Na-Metabisulfite and Na-EDTA were added to the containers to prevent urine sedimentation and oxidation. At the end of each 12-h interval, the patient aliquoted the urine in 2,5 ml polypropylene tubes (Eppendorf) and froze the urine samples at -20°C. Once a week, the frozen samples were brought to the laboratory where they were stored at -80°C until further analysis (no breaking of the cold chain). Moreover, at the end of each 12-h interval (at approx. 8 a.m. and approx. 8 p.m.), the patient completed the Daily Inventory of Activity, Routine and Illness (DIARI), a set of questionnaires including two 10 cm visual analogue scales (VAS) on perceived pain and RA disease activity as well as the short form of the *Eigenschaftswörterliste* (EWL), a German questionnaire that measures emotional states on three scales (mood, mental activity, irritation) (13). Furthermore, once a week, an in-depth interview was conducted with the patient via video call to determine the previous week’s emotionally meaningful positive and negative everyday incidents. Additionally, she had weekly appointments with her rheumatologist for blood sampling and to determine RA disease activity scores (total of 5 appointments). For further details on the study design, see (3).

### Subject description

The subject of this study was a 59-year-old Caucasian woman. She was married, had a 21-year-old son, and lived with her husband in Munich, the capital of Bavaria, in southern Germany. In 2005 (15 years prior to study start), she was diagnosed with Hashimoto’s thyroiditis, which was incidentally detected during a routine examination and was treated using a daily morning dose of thyroxine (37.5 mg). In 2015 (5 years prior to study start), she was diagnosed with RA. Additionally, three years before and up until study start the patient complained about having headaches and problems sleeping through. At study entry, her medication plan regarding RA consisted of a daily morning dose of prednisolone (1-2 mg) and sulfasalazine (2 g).

### Urine measurements

Urinary creatinine and neopterin concentrations were measured using High-Pressure Liquid Chromatography (HPLC) (Agilent 1100 system, Santa Clara, CA). Urinary cortisol (NT-DNOV010, Biomedica), ORM-2 (ABIN6969086), and IL-6 (DY206, R&D Systems) were determined using enzyme-linked immunosorbent assays (ELISA). Cortisol is the main effector of the hypothalamic-pituitary-adrenal (HPA) axis and has well described immunomodulatory properties (14). Neopterin is produced by monocytes and macrophages upon stimulation of T helper type 1 (Th1)-cell derived gamma-interferon (IFN-γ) and is used as a marker of non-specific systemic inflammation, for example in RA (15). Urinary levels represent neopterin biosynthesis well as it does not bind to receptors and undergoes rapid renal clearance (7). IL-6 is a pleiotropic cytokine with diverse functions in coordinating innate and adaptive immune responses (16). So far, there is no definitive answer on the proportionality of urine and serum IL-6 levels. Although the kidneys take part in the (urinary) elimination of IL-6 from systemic circulation with a pass ratio of 0.2% (6), urine and serum levels did not correlate in a cross-sectional analysis of spot samples (17). ORM-2 is an acute-phase reactant and its urinary levels have previously been identified as surrogates for assessing disease activity and prognosis of RA (18). For the determination of each of these biomarkers, all 60 consecutive urine concentrations were measured in one single run. Cortisol, neopterin, IL-6 and creatinine measurements were performed in duplicate, the mean values were used for further analysis. For some of the ORM-2 ELISA samples duplicate measurements were unavailable. Missing values were imputed with zero, while ORM-2 concentrations exceeding the highest value of the measuring range were imputed as 4000 pg/ml (= upper limit of the assay range). Urinary cortisol, neopterin, IL-6 and ORM-2 levels are expressed relative to urinary creatinine (per mol) to compensate for variations in urine density. As the use of creatinine to normalize urinary biomarkers has been questioned previously, we additionally considered 12-h urine excretion rates (amount of substance in test volume * total 12-h urine volume) for normalization (19).

### Rheumatic disease activity

Rheumatic disease activity over the study period was assessed using a combination of standard indices and patient-reported measures. Twice a day (at approx. 8 a.m. and approx. 8 p.m.), the patient completed subjective appraisals on RA disease activity and pain using 10 cm VAS (20). Moreover, once a week, the patient had an appointment with her rheumatologist in which the simplified disease activity index (SDAI) and the clinical disease activity index (CDAI) were determined. In addition, levels of C-reactive protein (CRP) in plasma and erythrocyte sedimentation rate (ESR) were analyzed to determine the disease activity index-28 score CRP (DAS28-CRP) and ESR (DAS28-ESR) (21, 22). The patient met her rheumatologist in the 12-h intervals 1, 10, 24, 38 and 52. Cut-off values for SDAI, CDAI and DAS28 to determine different RA disease activity states were used as proposed by (21).

### Statistical analysis

All statistical analyses were performed using *R* 4.2. Pearson product-moment correlations were conducted to determine the similarity between different normalization procedures of the urinary biomarkers. To investigate serial dependencies in the time series, autocorrelation functions (ACF) were calculated. Interrupted time series analyses (ITSA) were applied to evaluate differences between specific time series intervals. For this purpose, a type II ANCOVA model with lagged dependent variables was performed to control for autocorrelation, using the R package its.ancova [(23, 24)]. In all analyses, statistical significance was considered at *p* <.05.

## Results

**Figure 1:**
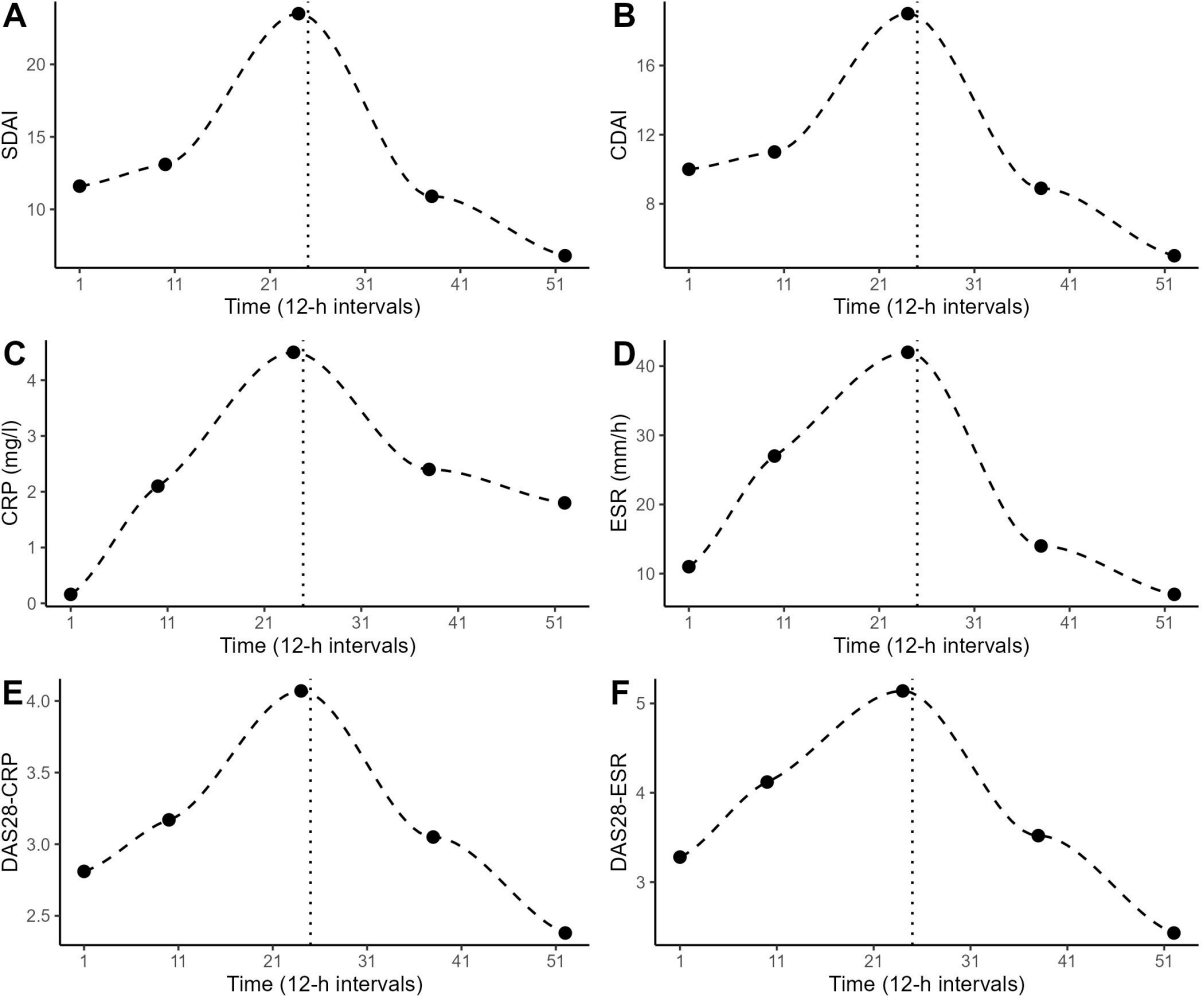
Weekly determinations of the patient’s RA disease activity by the rheumatologist at 12-h intervals 1, 10, 24, 38 and 52. The dashed line shows the LOESS curve. The dotted vertical line marks the timepoint of change in medication. **(A)** Simplified disease activity index (SDAI), **(B)** clinical disease activity index (CDAI), **(C)** c-reactive protein (CRP), **(D)** erythrocyte sedimentation rate (ESR), **(E)** disease activity score-28 (DAS28) using CRP, **(F)** DAS28 using ESR.

The patient experienced an increase in RA disease activity during the first 12 days of the study period as determined by SDAI, CDAI and DAS28-CRP/-ESR. Thereupon, her medication plan was adjusted (at 12-h interval number 25): the daily morning dose of prednisolone was elevated from 1-2 mg to 5 mg, sulfasalazine was deposed and a weekly injection of methotrexate (7.5 mg) was introduced (methotrexate injections were given at 12-h intervals 24, 38 and 52). All the weekly measures of RA disease activity that were assessed by the rheumatologist (CDAI, SDAI, CRP, ESR, DAS28-CRP/-ESR) showed a similar (inversed U-shaped) temporal pattern: an increase during the first 12 days of the study (until 12-h interval number 24) and a decline after the change in medication (**Figure 1**).

**Figure 2:**
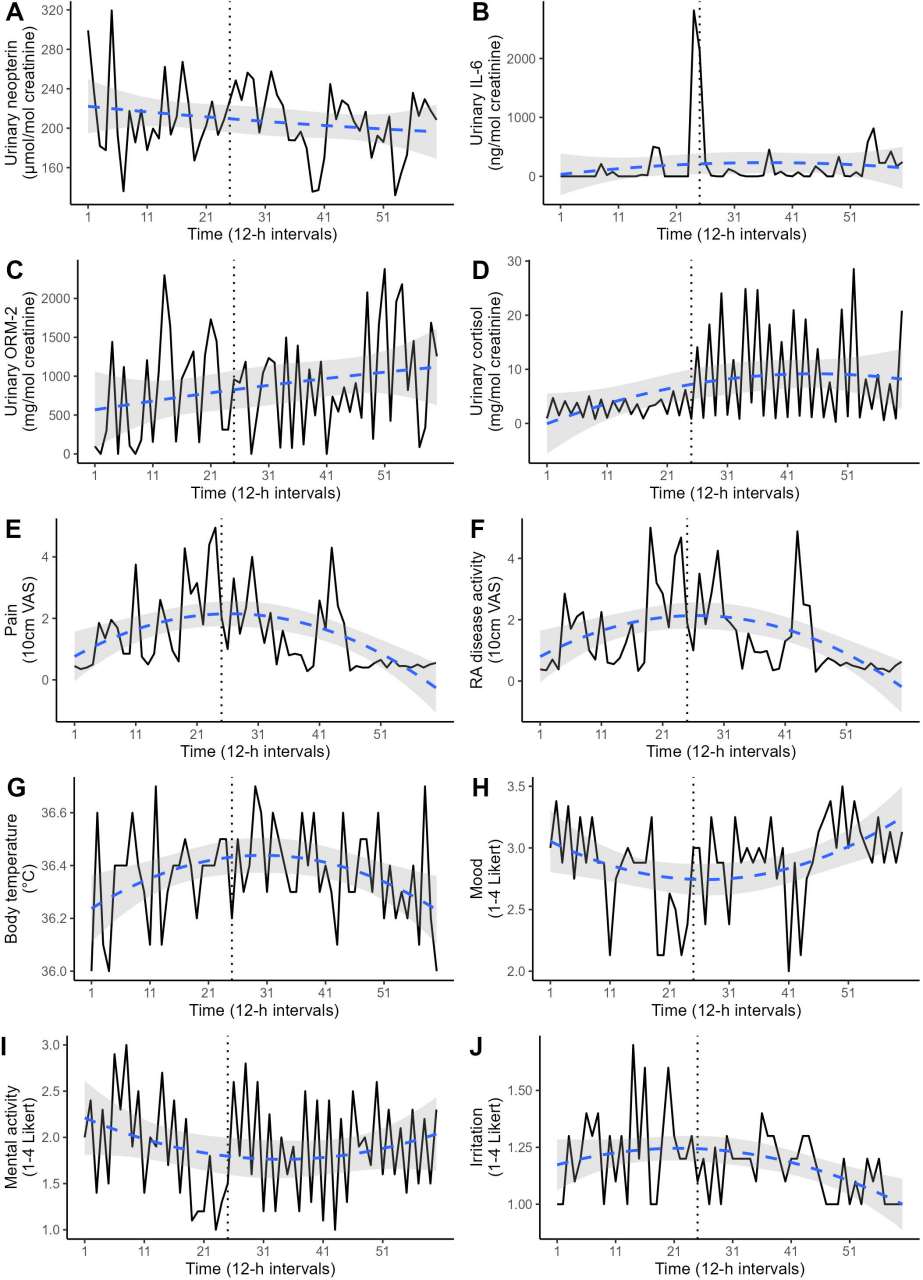
Various time series covering the 30-day study period in 12-h intervals (total: 60 measurements). The dotted vertical line marks the timepoint of change in medication. The dashed blue line represents the polynomial regression prediction, accompanied by its 95% confidence interval (gray area). **(A)** Urinary neopterin, **(B)** urinary interleukin-6 (IL-6), **(C)** urinary orosomucoid-2 (ORM-2), **(D)** urinary cortisol, **(E)** perceived pain, **(F)** perceived RA disease activity, **(G)** body temperature, **(H)** mood, **(I)** mental activity, **(J)** irritation.

Descriptive statistics on the consecutive urinary 12-h measurements (creatinine, cortisol, neopterin, IL-6, ORM-2) and the patient-reported outcomes (emotional states, pain, RA disease activity) are listed in **Table 1**, while their time-series are plotted in **Figure 2**. The resulting time series from the different normalization procedures were significantly correlated for each biomarker. For completeness, all the following statistical analyses were repeated using time series with 12-h urine excretion rates as alternatives to creatinine-normalized values, but no difference in the directions or significance of the results emerged (data not shown).

**Table 1:** Descriptive statistics regarding the levels of the 12-h urine analytes, body temperature and patient reports.

	Descriptive statistics			
	Mean	SD	Range	Unit
Creatinine				
Absolute 12-h excretion rate	4.35 4.55	± 1.59 ± 0.79	2.30 – 8.78 2.79 – 7.31	µmol/ml mmol/12h
Cortisol				
Absolute 12-h excretion rate Per creatinine	35.03 32.40 6.73	± 46.62 ± 39.34 ± 7.81	0.80 – 176.15 1.20 – 140.92 0.28 – 28.53	ng/ml μg/12h mg/mol
Neopterin				
Absolute 12-h excretion rate Per creatinine	906.53 940.67 207.84	± 369.81 ± 225.46 ± 36.78	442 – 2171 523.92 – 1882.80 131.91 – 319.46	pmol/ml nmol/12h µmol/mol
Interleukin-6				
Absolute 12-h excretion rate Per creatinine	0.78 786.88 179.26	± 2.13 ± 1964.70 ± 467.48	0 – 11.91 0 – 10539,31 0 – 2815.74	pg/ml pg/12h ng/mol
Orosomucoid-2				
Absolute 12-h excretion rate Per creatinine	3972.81 4.09 865.61	± 3212.20 ± 3.29 ± 667.24	0 – 8000 0 – 12 0 – 2378.31	ng/ml mg/12h mg/mol
Body function				
Body temperature	36.37	± 0.18	36 – 36.7	°C
Patient reports				
Mood Mental activity Irritation Pain RA disease activity	2.89 1.89 1.18 1.47 1.48	± 0.36 ± 0.54 ± 0.16 ± 1.24 ± 1.27	2 – 3.50 1 – 3 1 – 1.70 0.28 – 4.95 0.30 – 5	1-4 Likert 1-4 Likert 1-4 Likert 10cm VAS 10cm VAS

SD = standard deviation.

A significant negative ACF at lag1 was found in urinary cortisol levels (*r* = -.47, *p* < .01), indicating a circadian pattern with higher values during the day (12.05 ± 8.05 mg/mol creatinine) and lower values during the night (1.4 ± 0.77 mg/mol creatinine). Urinary neopterin, urinary IL-6 and urinary ORM-2 concentrations did not exhibit significant circadian rhythms.

Differences in the urinary 12-h measurements before (12-h intervals 1-24) and after (12-h intervals 25-60) the change in medication are shown in **Figure 2**. ITSA revealed that urinary cortisol levels increased significantly after the change (F = 49.37, *p* <.01). However, this increase was only evident for day levels (*F* = 15.00, *p* <.01), and not for night levels (*F* = 1.33, *p* = .30). Irritation also increased significantly after the medication change (F = 6.49, p = .01). The levels of neopterin (F = 0.54, *p* = .91), IL-6 (F = 0.96, *p* = .45), ORM-2 (F = 0.83, *p* = .43), perceived pain (*F* = 2.69, *p* = .10), perceived RA disease activity (*F* = 0.50, *p* = .71), mood (*F* = 2.12, *p* = .12), mental activity (*F* = 0.60, *p* = .85) and body temperature (*F* = 0.50, *p* = .71) did not differ before and after the medication change. A polynomial regression analysis was conducted to replicate the non-linear pattern in the disease activity indices (**Figure 1**). Both linear and quadratic time terms were fitted to the time series. Perceived pain, perceived RA disease activity, emotional states and body temperature exhibited a U-shaped pattern (**Figure 2**), mirroring the trends in disease activity indices seen in **Figure 1**. No such pattern was found for the time-series of urinary neopterin, urinary IL-6, urinary ORM-2 and urinary cortisol levels (**Figure 2**).

## Discussion

In this study, we monitored endocrine, immune and disease activity parameters in an RA patient over 30 days in 12-h intervals during a flare-up and subsequent change in medication ^1^. The urine samples generated in this study are quite unique as they allow the flare-up of RA disease activity and subsequent therapeutic response to be tracked continuously and cumulatively in a setting of high ecological validity. Multiple aliquots of each 12-interval’s urine collection are stored at –80°C. We would like to share this biobank with other researchers for further analysis and hypothesis-testing of the role of biomarkers in short-term changes of RA disease activity. During the study period, the RA disease state increased from low to high activity during the first 12 days (as measured by SDAI, CDAI and DAS28-CRP/-ESR). Thereupon, the medication plan was changed: the daily morning dose of prednisolone was elevated from 1-2 mg to 5 mg, sulfasalazine was deposed and a weekly injection of methotrexate (7.5 mg) was introduced. Within two weeks, this change in medication proved successful in reducing the RA disease activity toward scores considered to indicate low activity or remission (21) (**Figure 1**). Previously, CRP and ESR have been found to differ in their usefulness to monitor RA disease activity and treatment response, as CRP levels tend to drop quickly with treatment, while ESR levels can take weeks to normalize (22). Here, however, when applying naturalistic time series data, no difference was found in the responsive delays of CRP and ESR (**Figures 1C, D**).

Urinary cortisol levels increased significantly with the new medication regime but likely did not represent enhanced endogenous cortisol production but rather originated as a product of the ELISA-kit’s cross reactivity with prednisolone (46%; **Figure 2D**). However, only the diurnal levels of cortisol increased, whereas the nocturnal levels did not differ pre-post medication change, suggesting that the basal circadian rhythm was unaffected by this medication. The urinary assessments of immune system activity (neopterin, IL-6, ORM-2) were neither associated with the flare-up nor the change in medication, but instead remained stationary throughout the study period (**Figures 2A–C**). Only two decreases (12h-intervals 39 and 53) can be seen in the time series of neopterin after the change, which are likely explained by the newly introduced weekly methotrexate injections administered directly before these neopterin decreases during 12h-intervals 38 and 52.

These data indicate that urinary neopterin measurements are unrelated to short-term changes in RA disease activity and acute exacerbations. Such finding is consistent with a recent meta-analysis examining the role of neopterin in RA, which concludes that while neopterin levels are generally elevated in RA patients, no relation exists between specific neopterin levels and states of disease activity (25). This points to a difference in the biological pathways of RA-associated inflammation as measured by CRP and ESR, and neopterin-associated systemic inflammation. Since IL-6 and ORM-2 levels also did not correspond to the flare-up in our study, the same considerations might apply for these biomarkers as well. However, as IL-6 has previously been described as a crucial part of the immunological pathways of RA (16), the non-existent link between IL-6 and RA disease activity might also result from a limited comparability between serum and urine levels (17).

This study’s limitations emerge primarily from the described results being generated in a single RA patient, restricting their generalizability to the population. Moreover, assessment of neopterin, IL-6 and ORM-2 in the blood or synovial fluid of the patient in addition to the urinary measurements could have given more insights into their role and value as biomarkers. Also, the study focused on a narrow selection of biomarkers, excluding other potentially significant parameters (e.g., tumor necrosis factor-α [TNF-α], interleukin-1β [IL-1β]), which could be determined in further examinations. In addition, the usage of advanced time-series analysis, such as autoregressive integrated moving average (ARIMA) modeling, vector autoregression (VAR) models or time-varying modeling approaches (3, 26), could provide further insights into the temporal relations among variables in future studies.

In conclusion, this integrative single-case study clearly demonstrated the importance of process analysis for evaluating therapeutic measures in RA. In the studied patient, urinary neopterin, urinary IL-6 and urinary ORM-2 did not represent valid biomarkers for short-term changes in the disease activity of RA. Conversely and in accordance with previous reports (1, 3, 4, 20, 27), the results indicate that patient-reported outcomes and emotional states can be a useful tool in the ambulatory and longitudinal monitoring of disease activity, flare occurrence and treatment responses. Overall, this N-of-1 study highlights the benefit of frequently collecting psychological and immunological data over an extended period of time under conditions, which preserve the natural ebb and flow of daily life, with no external variability created by study-related guidelines or restrictions (3).

## Data Availability

The raw data supporting the conclusions of this article will be made available by the authors, without undue reservation.
